# Health-Related Quality of Life and Psychosocial Variables in Women with Colorectal Pelvic Floor Dysfunction: A Cross-Sectional Study

**DOI:** 10.3390/healthcare12060668

**Published:** 2024-03-15

**Authors:** Rocío Molina-Barea, Mahmoud Slim, Elena P. Calandre

**Affiliations:** 1 Department of Surgery, Hospital Universitario de Jaén, 23007 Jaén, Spain; rocio.molina.sspa@juntadeandalucia.es; 2 Instituto de Neurociencias, Universidad de Granada, 18016 Granada, Spain; mahmoud.slim@gmail.com; 3 Evidence Synthesis, Modelling & Simulation, Evidera, St. Laurent, Montreal, QC H4T1V6, Canada

**Keywords:** pelvic floor disorders, women, quality of life, anxiety, depression, dyssomnias, sexual behavior

## Abstract

Pelvic floor dysfunction comprises various disorders, including urinary incontinence, fecal incontinence, pelvic organ prolapse, and chronic pelvic pain. This study aimed to evaluate health-related quality of life (HRQoL), anxiety, depression, sleep disturbance, and sexual functioning in women with pelvic floor dysfunction of colorectal etiology compared with control women. Patients were recruited from a specialized colorectal unit and controls were selected from among the patients’ friends and relatives. Sociodemographic and clinical data were collected. Pelvic floor dysfunction distress and impact, HRQoL, depression, anxiety, insomnia, and sexual functioning were assessed using the following validated questionnaires: Short-Form Pelvic Floor Distress Inventory (PFDI-20), Short Form Pelvic Floor Impact Questionnaire (PFIQ-7), 36-Item Short-Form Health Survey (SF-36), Beck Depression Inventory II (BDI-II), Beck Anxiety Inventory (BAI), Insomnia Severity Index (ISI), and Changes in Sexual Functioning Scale (CSFQ). Statistical analyses included Welch’s *t*-test, Fisher’s exact test, and Spearman’s correlation coefficients. Eighty-four patients and 57 controls were included. Compared with controls, patients were more likely to be obese or overweight, have had higher numbers of deliveries, more vaginal deliveries, more frequent use of forceps, and have had more associated comorbidities, mainly in the urinary, neurological, and psychiatric domains. As expected, patients scored significantly higher than controls on both the PFDI-20 and PFIQ-7 and their respective sub-scales, with the highest mean values in the patient group on the sub-scales related to the colorectal–anal domain. QoL, depression, anxiety, insomnia, and sexual functioning were significantly worse in patients than in controls (*p* < 0.0001 in every case). In patients, PFIQ-7 scores correlated significantly with HRQoL (*p* < 0.001 for the physical component and *p* < 0.01 for the mental component), depression (*p* < 0.001), anxiety (*p* < 0.001), insomnia (*p* < 0.05), and sexual functioning scores (*p* < 0.05). Colorectal pelvic floor dysfunction had a markedly deleterious impact on the HRQoL, depression, anxiety, sleep disturbance, and sexual functioning of patients. It is concluded that colorectal pelvic floor dysfunction exerts a considerable burden on patients’ lives. Addressing these issues in clinical settings could significantly improve patients’ well-being.

## 1. Introduction

Pelvic floor dysfunction (PFD) is a common and frequently underdiagnosed pathology [1,2]. It includes different types of disorders such as urinary dysfunction, anorectal dysfunction, pelvic organ prolapse (POP), chronic pelvic pain, and sexual dysfunction [3]. These conditions frequently overlap [1,2]. Although pelvic floor dysfunction can be present in both sexes its prevalence is higher in women than in men due to the higher anatomical complexity of the female pelvic structures, as well as to the fact that pregnancy and delivery are well-established risk factors for its presentation [4,5]. Other relevant risk factors for PFD include aging [4,5,6] and obesity [4,5,7].

PFD of colorectal etiology includes disorders such as anorectal dysfunction, rectoceles, and some types of chronic pelvic pain. A rectocele is a form of POP that involves the herniation of the rectum through the rectovaginal septum into the posterior vaginal lumen, which frequently causes anal incontinence and/or dyspareunia. Its prevalence is estimated at 30–50% of multiparous women older than 50 years [8]. Anorectal dysfunction includes fecal incontinence (FI), i.e., the involuntary loss of feces and/or flatus, and chronic functional constipation or dyssynergic defecation, usually associated with excessive straining and/or feeling of incomplete evacuation. The worldwide prevalence of fecal incontinence has been estimated to be 8%, and, specifically in Europe, 6.5%. It is more frequent in women than in men (9.1% vs. 7.4%, OR: 1.17, *p* < 0.001) and in older than in younger people (9.3% vs. 4.9% in people aged ≥60 years and <60 years, OR:1.75, *p* < 0.001) [9]. There are no definite data concerning the prevalence of fecal incontinence in Spain, as acknowledged by the Spanish Coloproctological Society [10]. The prevalence of chronic functional constipation has been estimated to affect 10% to 15% of the population, depending on the Rome criteria applied for its diagnosis, and it has also been shown to be higher in women than men [11]. Data concerning dyssynergic defecation are less accurate; it is usually accepted that between 27% and 50% of cases of chronic constipation are due to dyssynergia [12,13]. Similarly to fecal incontinence, it is more frequent in women and in older people [12,13]. Chronic pelvic pain is estimated to affect up to 24% of women worldwide, and its most frequent colorectal etiology is levator syndrome [14].

Both urinary dysfunction and POP have been extensively investigated in women. In contrast, anorectal dysfunction has rarely been investigated, probably because of its lower prevalence in relation to the other two conditions [4].

For instance, in a targeted literature search conducted by the study authors using PubMed at the time of drafting this article, we identified 213 papers published in 2023 related to “incontinence” in women, of which 199 dealt with urinary incontinence, while only 13 addressed fecal incontinence and just one covered both types. Despite the considerable distress caused by anorectal dysfunction, only a few studies have documented the substantial impairment in quality of life experienced by women with such dysfunction [11,12]. Frequently, due to the embarrassment that they feel related to their symptoms they do not even mention them to their physician [15,16]. Thus, it is important to study the burden associated with anorectal dysfunction.

The aim of the present study was to evaluate health-related quality of life, anxiety, depression, sleep quality, and sexual functioning in women with pelvic floor dysfunction of colorectal etiology compared to control subjects.

## 2. Patients and Methods

### 2.1. Study Design

This was a cross-sectional study performed with patients attending pelvic floor consultations at the Unidad de Coloproctología del Complejo Hospitalario de Jaén (Spain) and with control subjects. The study protocol was approved by the Human Research Ethics Committee of the hospital according to the 2013 amendment of the 1964 Helsinki declaration. Written informed consent was required from all study participants.

### 2.2. Participants

The patient group included women aged ≥18 years with a prior diagnosis of a pelvic floor disorder of colorectal etiology for at least the last three months. Exclusion criteria included the presence of a concomitant severe neurological, mental, pulmonary, cardiovascular, renal and/or hepatic disease, and/or being unable to fully understand or complete the study questionnaires.

Controls were recruited among those patients’ relatives and/or friends that accompanied them to the hospital visits. This was in order to ensure that subjects in this group shared similar socioeconomic backgrounds and age groups with the patients. The control group included women aged ≥18 years who had not previously been diagnosed by a physician with any pelvic floor disorder. Exclusion criteria were the same as those of the patient group. Both patients and controls experiencing associated comorbidities that allowed them to have a normal life, such as hypertension, migraine, etc., were allowed to participate.

One of the study investigators was responsible for contact with patients as well as with controls. She informed them about the objectives of the study, that their participation would be voluntary, that loss of interest in participating would not influence the medical care they received, and that the collected data would be anonymous. She explained the content of the Case Report Forms (CRFs) that included the different questionnaires and, in the case of controls, also questions regarding their demographic and clinical backgrounds. She gave the CRFs to each patient or control participant and asked them to return the forms on the same day to avoid the possibility of losing data.

### 2.3. Primary Outcome Measures

1. Pelvic floor burden—The Short Form of the Pelvic Floor Distress Inventory (PFDI-20): This is a self-administered 20-item questionnaire which produces a summary score and encompasses three sub-scales: the 6-item Urinary Distress Inventory (UDI-6), the 6-item Pelvic Organ Distress Inventory (POPDI-6), and the 8-item Colorectal–Anal Distress Inventory (CRADI-8). Each sub-scale score ranges from 0 to 100 and the total score is calculated by adding the three sub-scales scores. Higher scores are indicative of greater distress [17].
2. Pelvic floor impact—The Short Form of the Pelvic Floor Impact Questionnaire (PFIQ-7): This is a self-administered 7-item questionnaire that assesses how the bladder, bowel, or vaginal symptoms separately impact functioning. It encompasses three sub-scales: the 7-item Urinary Impact Questionnaire (UIQ-7), the 7-item Colorectal–Anal Impact Questionnaire (CRAIQ-7), and the 7-item Pelvic Organ Prolapse Impact Questionnaire (POPIQ-7). Each sub-scale score ranges from 0 to 100 and the overall summary score is obtained by adding the total scores of the three sub-scales. Higher scores are indicative of greater impact [17].

We used the Spanish-validated versions of the PFDI-20 and PFIQ-7 [18].

### 2.4. Secondary Outcome Measures

1. Health-related quality of life—The 36-Item Short-Form Health Survey (SF-36): This is self-reported questionnaire that is widely used to evaluate health-related quality of life. Its 36 items cover the following eight domains: Physical Functioning (PF), Role Physical (RP), Bodily Pain (BP), General Health (GH), Vitality (VT), Social Functioning (SF), Role Emotional (RE), and Mental Health (MH). These can be summarized in two components: Physical Component Summary (PCS), and Mental Component Summary (MCS). The score for each dimension ranges from 0 to 100, with higher scores indicating better performance in each domain [19]. We used the Spanish-validated version [20].
2. Depression—The Beck Depression Inventory II (BDI-II): The BDI-II is a self-reported questionnaire consisting of 21 items. Total scores range from 0 to 63, with higher scores indicating more severe symptomatology. Range values of 0–13 are considered minimal depression, 14–19 indicate mild depression, 20–28 suggest moderate depression, and 29–63 indicate severe depression [21]. We used the Spanish-validated version [22].
3. Anxiety—The Beck Anxiety Inventory (BAI): The BAI is a self-reported questionnaire consisting of 21 items. Total scores range from 0 to 63, with higher scores indicating more severe symptomatology. Scores of 0–9 are considered normal, 10–18 indicate mild to moderate anxiety, 19–29 suggest moderate to severe anxiety, and 30–63 indicate severe anxiety [23]. We used the Spanish-validated version [24].
4. Sleep disturbance—The Insomnia Severity Index (ISI): The ISI is a self-reported questionnaire consisting of 7 items. Total scores range from 0 to 28, with higher scores indicating more severe sleep problems. Scores of 0–7 are considered normal, 8–14 indicate subthreshold insomnia, 15–21 suggest moderate insomnia, and 22–28 indicate severe insomnia [25]. We used the Spanish-validated version [26].
5. Sexual behavior—The Changes in Sexual Functioning Scale (CSFQ): The CSFQ is a self-reported questionnaire comprising 14 items for females and 14 items for males. Total scores range from 14 to 70, with cut-off points of 41 for females and 47 for males, where lower values indicate sexual dysfunction. It has five sub-scales that specifically measure the following domains: sexual desire/frequency, sexual desire/interest, sexual pleasure, sexual arousal/excitement, and sexual orgasm/completion [27]. We used the Spanish-validated questionnaire [28].

### 2.5. Sample Size and Statistical Analysis

The sample size was determined using G*Power version 3.1.9.6, with an alpha level of 0.05 and an anticipated effect size of 0.8 for the PFDI-20 [17]. A minimum of 42 subjects per group was necessary to potentially detect a statistically significant difference between patients and controls with a power of 95%. Given our objective to evaluate the differences in multiple outcomes of interest, we enrolled a slightly higher sample size per group.

Continuous data were described using means and standard deviations, and comparisons between patients and controls were analyzed using Welch’s *t*-test. Categorical data were described using the absolute and relative frequencies, and comparisons between patients and controls were analyzed using Fisher’s exact test. A *p*-value < 0.05 was considered to be statistically significant. Spearman nonparametric correlation coefficients were used to evaluate the relationships between variables.

The statistical analysis was performed using GraphPad Prism, Version 9.5.1 (GraphPad Software, San Diego, CA, USA).

## 3. Results

The total sample included 84 patients and 57 controls. Among patients, 42 (50%) suffered fecal incontinence, 24 (28.6%) dyssynergic defecation, 12 (14.3%) rectocele, and six (7.1%) chronic pelvic pain due to hypertonicity of the elevator muscle of the anus. Although none of them have consulted a physician for these reasons, when answering about suffering any chronic disease, 10 (17.5%) control women reported chronic constipation, three (5.3%) pelvic organ prolapse, and two (3.5%) chronic pelvic pain.

Sociodemographic and clinical characteristics are shown in Table 1. Although both samples were generally comparable, there were significant differences regarding the body mass indexes, with higher proportions of overweight and obese participants in the patient group.

Table 2 shows the obstetric backgrounds and associated comorbidities; significant differences were found between both groups regarding the number of children, the delivery types, and the associated pathologies. In relation to their obstetric background, patients had higher numbers of children, smaller numbers of cesarian sections, and were more likely to have experienced at least an instrumental delivery using forceps rather than a vacuum. Regarding the concomitant pathologies, the most striking differences were found in neurological diseases (with a higher number of patients experiencing migraines), urinary diseases (with a higher number of patients experiencing urinary incontinence), and psychiatric diseases (with a higher number of patients experiencing mixed anxious–depressive state).

Table 3 shows that patients scored significantly higher than controls on both scales and their respective sub-scales. As expected, the highest mean values in the patient group were found in the sub-scales related to the colorectal–anal domain. 

As shown in Table 4, patients scored significantly higher than controls in all of the SF-36 domains with the exception of the General Health and Mental Health domains, where differences between patients and controls were not statistically significant.

Depression and anxiety scores were also significantly higher in patients than in the controls (Table 5). Twenty-nine (50.1%) controls and 58 (69.0%) patients had anxiety scores ≥10, indicating clinically relevant anxiety, and 20 (35.1%) controls and 53 (63.1%) patients had depression scores ≥14, indicating clinically relevant depression. In the control group, mean depression and anxiety scores were within the normal ranges, whereas those of the patients were in the ranges suggesting moderate depression and anxiety. Additionally, significantly worse ISI scores were observed in the patient group, with mean scores indicating subthreshold insomnia (Table 5); 33 (57.9%) controls and 60 (71.4%) patients showed scores ≥8, indicating clinically relevant insomnia.

Sexual functioning was clearly impaired in the patient group (Table 5), with the mean total CSFQ scores well below the 41 cut-off point established to delimitate sexual dysfunction in women. This was not the case in the control group.

As can be seen in Table 6A, there were significant correlation coefficients between the PFIQ-7 total scores and every secondary outcome variable, whereas in the case of the PFDI-20 the only significant correlation was with SF-36 PCS.

## 4. Discussion

The main finding of our study is that pelvic floor dysfunction of colorectal etiology inflicts a considerable psychosocial burden on patients. This condition is associated with a marked decline in health-related quality of life, as well as increased depressive symptomatology, anxiety, sleep problems, and sexual dysfunction. An additional unexpected finding was that a non-negligible number of women in the control group also experienced some form of pelvic floor dysfunction that they did not disclose to any healthcare professional. This was probably related to the well-known fact that women with PFD frequently do not consult with their physician for fear of disclosing symptoms that they perceive as embarrassing [29,30,31,32].

As expected, PFDI-20 total scores were higher than those found in the general Spanish population [33]. Although similar to those found in other patients with pelvic floor dysfunction [17], patients in our study showed higher mean CRADI-8 scores than the former. This could be explained by the fact that our sample specifically consisted of women with pelvic floor dysfunction of colorectal–anal etiology. Furthermore, the mean PFIQ-7 scores and CRAIQ-7 sub-scale scores were markedly higher than those found by Barber et al. (2005) [17]. Comparing our data with those of a study that investigated utility scores in women with fecal incontinence [34], we found that our mean PFDI-20 and CRADI-8 scores were roughly similar. However, once again, the mean PFIQ-7 and CRAIQ-7 scores for our sample were much higher than those reported in the former study [34]. As both comparison studies [17,34] were conducted with U.S. women and our study was conducted with Spanish women, it could be hypothesized that perhaps the differences in the impact of pelvic floor dysfunction could be related to cultural differences.

Our results emphasize the relevance of body weight as a risk factor for pelvic floor dysfunction, with 65.5% of the patients being either overweight or obese (body mass index [BMI] ≥ 25 kg/m^2^) compared to only 38.6% of women in the control group. A review article about obesity and pelvic floor dysfunction confirmed the positive relationship between increased BMI and fecal dysfunction, as well as the improvement of the latter following weight loss [35].

Our data are also consistent with the role that obstetric data play as potential risk factors for pelvic floor dysfunction. Compared to the control group in our study, patients had higher numbers of children, more vaginal deliveries, and more frequent use of forceps than of vacuums when having experienced an instrumental delivery. Multi-parity as well as vaginal birth are acknowledged risk factors for pelvic floor dysfunction [5], and forceps delivery has been found to be associated with higher risk of POP and pelvic floor muscle trauma compared to vacuum delivery [36].

Patients also presented a higher number of associated comorbidities than control women. Differences were particularly marked in the case of psychiatric comorbidities, neurological comorbidities, and renal/urinary comorbidities. The most frequent psychiatric comorbidity was mixed anxiety and depression disorder, a finding not surprising as both anxious and depressive symptomatology have been reported both in women and in men with pelvic floor dysfunction [37]. Regarding comorbid neurological diseases, eleven (13.1%) patients experienced headache, a complaint that was not found among controls. In terms of comorbid urinary symptomatology, 17 (20.2%) patients in our study reported urinary incontinence versus none in the control group. These findings align with the already established association found between fecal and urinary incontinence, as reported in previous studies [1,2].

We found that all the domains of the SF-36 questionnaire were significantly lower in patients than in controls. In a study performed by Peinado-Molina et al. (2023) [38] in women from the general population, the authors evaluated health-related quality of life by using the SF-12, a shorter version of the SF-36. In line with our study’s findings, they reported significantly lower scores on all eight domains in women with urinary incontinence, fecal incontinence, prolapse, or pelvic pain compared to women without these conditions [38]. Among those with any pelvic floor disorder, the worst scores were seen in those with fecal incontinence. Compared to the scores reported by Peinado-Molina et al. [38], our patients had relatively lower scores, which could be attributed to the fact that their sample was drawn from the general population, whereas ours was recruited in a tertiary hospital setting.

Anxiety and/or depression in patients with pelvic floor disorders have been mainly evaluated in patients with overactive bladder syndrome, with limited information available regarding other pelvic floor disorders. A study conducted in 1510 patients attending a pelvic care center found that the prevalence of clinically relevant anxiety was 30.9% and the prevalence of clinically relevant depression was 20.3% [37]. In our study, these percentages were markedly higher, both in patients and in controls. The strikingly high prevalence of anxiety and depressive symptomatology among control women is noteworthy, but these numbers were clearly surpassed by those in the patient group, confirming that anxiety and depression are common in patients with pelvic floor disorders of colorectal etiology.

In relation to sleep quality, we only found one other study that evaluated this factor in the context of pelvic floor dysfunction, but the authors of this study only examined the influence of sleep quality on quality of life without assessing its correlation with pelvic floor dysfunction [38]. Data from our study showed that a noticeable proportion of both patients and controls showed clinically relevant sleep disturbances. Again, similar to anxiety and depression scores, the prevalence of sleep problems was relatively high in control women; however, it was even higher in the patient group.

Sexual functioning has been shown to be impaired in women with pelvic floor disorders. A narrative review published in 2019 reported that, whereas studies conducted in the general population found a prevalence of sexual dysfunction in females ranging from 30% to 50%, studies conducted in women with pelvic floor disorders found a higher prevalence, ranging from 50% to 83% [39]. This review also found that women with fecal incontinence reported higher rates of sexual dysfunction than those with urinary incontinence. In our sample, the sexual dysfunction was found to be remarkably higher among patients, with every domain of sexual function being impaired compared to the control group (Table 5). Martínez-Galiano et al. (2023) [40] found a prevalence of 28.6% for sexual dysfunction in a sample of 1008 women. Furthermore, their study showed that women with sexual dysfunction had significantly higher mean scores in the UDI-6, POPDI-6, and CRADI-8 sub-scales of the PFDI-20 than those without sexual dysfunction [40].

In the patient group of our study, we found significant correlations between the PFIQ-7 scores and the scores of every secondary outcome measure, but not between the PFDI-20 scores and the secondary outcome measures. The lack of significant correlations with the PFDI-20 may be attributed to the nature of the questionnaire itself. The authors of the PFDI and PFIQ, and their shorter versions, envisaged both questionnaires as two complementary instruments for evaluating the quality of life of women with pelvic floor disorders [17]. However, whereas the PFIQ-7 questions are specifically focused on the “life impact” caused by urinary, rectal, and vaginal symptoms, the questions on the PFDI-20 are focused on “symptoms distress” and whether the symptoms are more or less bothersome for the patient. This distinction in the underlying construct of the two questionnaires may potentially explain the presence of statistically significant correlations with the PFIQ-7 and not with the PFDI-20. In particular, the statistically significant correlations between the PFIQ-7 and the psychosocial and quality of life outcomes in our study further emphasize its validity in capturing the “life impact” of the disease. On the other hand, the PFDI-20 can be seen more as a questionnaire that measures the presence of symptoms and intensity of symptoms. Therefore, given its reliance on the symptomatic status of the patient, the PFDI-20 may not fully capture the multidimensional impact of pelvic floor disorders, leading to a lack of significant correlations with the secondary outcomes. This explanation is further supported by the fact that the PFDI-20 only correlated with the physical component of the SF-36 in our study.

The main strength of our study is that, to the best of our knowledge, it is the first study to assess, both health-related quality of life and psychosocial variables in women with pelvic dysfunction of colorectal etiology. Several limitations, however, must be acknowledged. The main one is that the cross-sectional design is prone to reverse causality [41], i.e., we cannot ascertain if the diminished quality of life, depressive and anxious symptomatology and sleep disturbance are a result of the pelvic floor disorder. Also, as our sample was drawn from a tertiary hospital setting, the generalizability of our findings to the general population may be limited. Patients are transferred from their family physician to a specialist (urologist, gynecologist or gastroenterologist) consultation, and from there to the tertiary attention unit; thus, only the most severely affected patients are seen in our unit.

## 5. Conclusions

Notwithstanding the afore mentioned limitations, we believe that our results highlight the considerable burden of colorectal pelvic floor disorders on various aspects of patients’ lives in terms of decreased quality of life, anxiety, depression, insomnia, and impairment of sexual functioning. These findings indicate the need for comprehensive attention to these patients, considering not only their physical but also their mental well-being. While medical and surgical patients’ needs are well attended, they are often not referred to pain clinics or the mental health care units. Protocols for better assistance for patients with colorectal PFD should be developed.

## Figures and Tables

**Table 1:** Sociodemographic data of the participants.

	Patients (N = 84)	Controls (N = 57)	*p* Value
Age (years) [mean ± s.d.]	50.5 ± 10.8	50.4 ± 13.6	0.967
Body Mass Index (BMI) [N(%)]			
Normal (18.5–24.9)	20 (23.8)	35 (61.4)	0.0003
Overweight (25–29.9)	34 (40.5)	15 (26.3)	
Obesity (≥30)	21 (25.0)	7 (12.3)	
Missing data	9 (10.7)		
Marital status [N(%)]			
With partner	73 (86.9)	53 (92.9)	0.762
Without partner	7 (8.3)	4 (7.0)	
Missing data	4 (4.8)		
Educational status [N(%)]			
Primary school	41 (48.8)	19 (33.3)	0.059
Secondary school	19 (22.6)	19 (33.3)	
University	16 (19.0)	19 (33.3)	
Missing data	8 (9.5)		
Employment status [N(%)]			
Not working	34 (40.5)	21 (36.8)	0.379
Working	42 (50.0)	36 (63.3)	
Missing data	8 (9.5)		

Fisher’s exact test was performed only with available data; missing data were not considered. *p*-value was <0.05 (significance threshold), indicating that the difference between patients and controls was statistically significant.

**Table 2:** Obstetric backgrounds and associated comorbidities.

	Patients (N = 84)	Controls (N = 57)	*p* Value *
Number of children [N(%)]			
0	5 (5.9)	18 (31.6)	0.0003
1–3	55 (65.5)	38 (66.7)	
≥4	8 (9.5)	1 (1.7)	
Missing data	16 (19.0)		
Delivery type [N(%)]			
Vaginal	102 (91.9)	49 (72.1)	0.0006
Cesarean section	9 (8.1)	19 (27.9)	
At least one instrumental delivery [N(%)]			
No	31 (38.1)	22 (38.6)	<0.0001
Yes: forceps	21 (25.0)	4 (7.1)	
Yes: vacuum	2 (2.4)	12 (21.1)	
Missing data	30 (35.7)	19 (33.3)	
Comorbidities:			
Cardiovascular	15 (17.8)	7 (12.3)	0.0001
Gastrointestinal	15 (17.8)	4 (26.3)	
Metabolic	21 (25.0)	8 (14.0)	
Musculoskeletal	26 (30.1)	12 (21.1)	
Neurological	20 (23.8)	1 (1.8)	
Neoplastic	5 (5.9)	0	
Renal/urinary	19 (22.6)	1 (1.8)	
Respiratory	9 (10.7)	7 (12.3)	
Psychiatric	25 (29.8)	5 (8.8)	

* Fisher’s exact test was performed only with available data; missing data were not considered. *p*-value was <0.05 (significance threshold), indicating that the difference between patients and controls was statistically significant.

**Table 3:** Primary outcome variables: PFDI-20 and PFIQ-7.

	Patients (N = 84)	Controls (N = 57)	*p* Value *
PFDI-20 total scores	118.3 ± 58.5	47.7 ± 40.3	<0.0001
	(14.6–279.9)	(0–169.8)	
UDI-6	37.8 ± 25.8	21.9 ± 16.7	<0.0001
	(0–100)	(0–58.3)	
POPDI-6	34.5 ± 26.2	15.4 ± 16.1	<0.0001
	(0–108.3)	(0–54.2)	
CRADI-8	58.1 ± 17.5	15.1 ± 16.1	<0.0001
	(12.5–96.9)	(0–65.6)	
PFIQ-7 total scores	112.3 ± 74.2	35.3 ± 37.9	<0.0001
	(0–295)	(0–152.4)	
UIQ-7	29.9 ± 30.8	13.2 ± 17.8	<0.0001
	(0–100)	(0–57.1)	
POPIQ-7	28.9 ± 32.8	9.4 ± 17.6	<0.0001
	(0–100)	(0–100)	
CRAIQ-7	55.9 ± 31.2	12.6 ± 15.7	<0.0001
	(0–100)	(0–61.9)	

First line values include mean ± s.d. and second line values include the range of minimum and maximum values. * *p*-value was <0.05 (significance threshold), indicating that the difference between patients and controls was statistically significant. PFDI: Pelvic Floor Distress Inventory; UDI-6: Urinary Distress Inventory; POPDI-6: Pelvic Organ Distress Inventory; CRADI-8: Colorectal-Anal Distress Inventory; PFIQ-7: Pelvic Floor Impact Questionnaire; UIQ-7: Urinary Impact Questionnaire; POPIQ-7: Pelvic Organ Prolapse Impact Questionnaire; CRAIQ-7: Colorectal–Anal Impact Questionnaire.

**Table 4:** Secondary outcome variables: SF-36 Health Survey.

	Patients (N = 84)	Controls (N = 57)	*p* Value *
PF (Physical Functioning)	56.9 ± 27.6	89.7 ± 11.3	<0.0001
	(0–100)	40–100	
RP (Role Physical)	36.1 ± 42.8	79.9 ± 23.1	<0.0001
	(0–100)	(0–100)	
BP (Bodily Pain)	41.4 ± 29.2	67.1 ± 26.4	<0.0001
	(0–100)	(0–100)	
GH (General Health)	43.3 ± 22.9	45.5 ± 14.6	0.0994
	(0–95)	(10–75)	
VT (Vitality)	37.6 ± 22.8	51.3 ± 14.4	<0.0001
	(0–95)	(25–80)	
SF (Social Functioning)	46.1 ± 28.6	73.7 ± 20.6	<0.0001
	(0–100)	(12.5–100)	
RE (Role Emotional)	36.5 ± 43.8	63.2 ± 27.9	<0.0001
	(0–100)	(0–100)	
MH (Mental Health)	47.9 ± 25.8	56.8 ± 14.5	0.0104
	(0–96)	(8–92)	
PCS (Physical Component Summary)	38.2 ± 11.1	51.0 ± 5.15	<0.0001
	(20–60.8)	(33.8–60.6)	
MCS (Mental Component Summary)	33.5 ± 10.4	36.9 ± 7.2	0.0261
	(14.2–57.6)	(13.6–49.9)	

First line values include mean ± s.d. and second line values include the range of minimum and maximum values. * *p*-value was <0.05 (significance threshold), indicating that the difference between patients and controls was statistically significant.

**Table 5:** Secondary outcome variables: depression, anxiety, sleep disturbance, and sexual behavior.

	Patients (N = 84)	Controls (N = 57)	*p* Value *
BDI total scores	20.3 ± 14.1	10.8 ± 9.3	<0.0001
	(0–50)	(0–35)	
BAI total scores	19.5 ± 15.2	10.5 ± 7.3	<0.0001
	(0–57)	(0–32)	
ISI total scores	14.3 ± 8.3	8.0 ± 4.9	<0.0001
	(0–28)	(0–20)	
CSFQ total scores	37.5 ± 11.3	43.9 ± 9.5	<0.0001
	(22–66)	(22–66)	
Desire/frequency	5.1 ± 2.0	6.4 ± 1.4	<0.0001
	(2–9)	(2–9)	
Desire/interest	5.7 ± 2.3	7.2 ± 2.2	<0.0001
	(3–13)	(3–13)	
Pleasure	2.4 ± 1.2	3.4 ± 0.9	<0.0001
	(1–5)	(2–5)	
Arousal/excitement	7.7 ± 3.3	9.8 ± 2.6	<0.0001
	(3–15)	(3–15)	
Orgasm/completion	8.1 ± 3.7	11.2 ± 2.8	<0.0001
	(3–15)	(3–15)	

First line values include mean ± s.d. and second line values include the range of minimum and maximum values. * *p*-value was <0.05 (significance threshold), indicating that the difference between patients and controls was statistically significant. BDI-II: Beck Depression Inventory; BAI: Beck Anxiety Inventory; ISI: Insomnia Severity Index; CSFQ: Changes in Sexual Functioning Scale.

**Table 6:** (**A**): Spearmen correlation coefficients among outcome variables in patients. (**B**): Spearmen correlation coefficients among outcome variables in controls.

(A)							
Patients	2	3	4	5	6	7	8
1. PFDI-20	0.464 ***	−0.215 *	−0.169	0.122	0.162	0.023	−0.040
2. PFIQ-7	—	−0.598 ***	−0.346 **	0.041 ***	0.385 ***	0.268 *	−0.269 *
3. SF-36 PCS	—	—	0.558 ***	−0.685 ***	−0.718 ***	−0.404 ***	0.412 ***
4. SF-36 MCS	—	—	—	−0.769 ***	−0.719 ***	−0.550 ***	0.317 **
5. BDI-II	—	—	—	—	0.877 ***	0.703 ***	−0.339 **
6. BAI	—	—	—	—	—	0.616 ***	−0.459 ***
7. ISI	—	—	—	—	—	—	−0.316 **
8. CSFQ	—	—	—	—	—	—	—
**(B)**							
**Controls**	**2**	**3**	**4**	**5**	**6**	**7**	**8**
1. PFDI-20	0.784 ***	−0.138	0.106	0.302 *	0.348 **	0.267 *	−0.023
2. PFIQ-7	—	−0.286 *	−0.055	0.344 **	0.421 **	0.341 **	−0.209
3. SF-36 PCS	—	—	−0.182	−0.020	−0.052	0.006	−0.121
4. SF-36 MCS	—	—	—	0.021	0.038	−0.038	0.261 *
5. BDI-II	—	—	—	—	0.832 ***	0.747 ***	−0.323 *
6. BAI	—	—	—	—	—	0.808 ***	−0.322 *
7. ISI	—	—	—	—	—	—	−0236
8. CSFQ	—	—	—	—	—	—	—

*p*-value was <0.05 (significance threshold), indicating that the difference between patients and controls was statistically significant. ***: *p* < 0.001; **: *p* < 0.01; *: *p* < 0.05. PFDI: Pelvic Floor Distress Inventory; PFIQ: Pelvic Floor Impact Questionnaire; SF-36 PCS: Short-Form Health Survey Physical Component Summary; SF-36 MCS: Short-Form Health Survey Mental Component Summary; BDI-II: Beck Depression Inventory II; BAI: Beck Anxiety Inventory; ISI: Insomnia Severity Index; CSFQ: Changes in Sexual Functioning Scale.

## Data Availability

The data that support the findings of the study are available upon request from the corresponding author. The data are not publicly available due to privacy and ethical restrictions.
